# Resveratrol prevents the release of neutrophil extracellular traps (NETs) by controlling hydrogen peroxide levels and nuclear elastase migration

**DOI:** 10.1038/s41598-024-59854-2

**Published:** 2024-04-20

**Authors:** Thayana Roberta Ferreira de Mattos, Marcos Antonio Formiga-Jr, Elvira Maria Saraiva

**Affiliations:** Laboratório de Imunidade Inata, Departamento de Imunologia, Instituto de Microbiologia Paulo de Góes, Universidade Federal do Rio de Janeiro (UFRJ), Rio de Janeiro, RJ Brazil

**Keywords:** Neutrophil extracellular traps, Elastase, Resveratrol, Immunology, Inflammation, Innate immune cells

## Abstract

Neutrophil extracellular traps (NETs) are defense mechanisms that trap and kill microorganisms and degrade cytokines. However, excessive production, dysregulation of suppression mechanisms, or inefficient removal of NETs can contribute to increased inflammatory response and the development of pathological conditions. Therefore, research has focused on identifying drugs that inhibit or delay the NET release process. Since reactive oxygen species (ROS) play a significant role in NET release, we aimed to investigate whether resveratrol (RSV), with a wide range of biological and pharmacological properties, could modulate NET release in response to different stimuli. Thus, human neutrophils were pretreated with RSV and subsequently stimulated with PMA, LPS, IL-8, or *Leishmania*. Our findings revealed that RSV reduced the release of NETs in response to all tested stimuli. RSV decreased hydrogen peroxide levels in PMA- and LPS-stimulated neutrophils, inhibited myeloperoxidase activity, and altered the localization of neutrophil elastase. RSV inhibition of NET generation was not mediated through A2A or A2B adenosine receptors or PKA. Based on the observed effectiveness of RSV in inhibiting NET release, our study suggests that this flavonoid holds potential as a candidate for treating NETs involving pathologies.

## Introduction

Neutrophil extracellular traps (NETs) are web-like structures primarily composed of a DNA scaffold associated with several proteins^1^. NETs are an essential immune defense mechanism capable of trapping, thus preventing systemic spread and killing invading microorganisms as well^1–7^. Different cell types release these traps, which are also highly conserved among organisms^8,9^. The molecular targets guiding the production of NETs depend on the stimulus presented to neutrophils, which can include bacteria, viruses, fungi, parasites, cytokines, laminin, and PMA^1–7,10^. Despite the diversity of signaling intermediates, hydrogen peroxide (H_2_O_2_) appears to play a crucial role, as it facilitates the movement of two azurophilic granule-resident enzymes, elastase (NE) and myeloperoxidase (MPO), towards the nucleus, initiating the process of chromatin decondensation^11^.

However, numerous studies have demonstrated that excessive production, dysregulation of suppression mechanisms, and inefficiency of NETs removal can be detrimental to tissues, leading to increased toxicity and inflammation, thereby exacerbating diseases such as sepsis, atherosclerosis, autoimmune diseases, cancer, and COVID-19^3,12–14^. DNAse treatment is a commonly used option^15^, but it is important to note that it is only effective after the release of NETs. Moreover, DNAse only breaks down DNA and does not affect harmful histones or granular proteins that can damage cells^13^. Therefore, it is essential to identify drugs targeting the active stage before NET release to mitigate or eliminate the damage caused by these structures.

Antioxidants have been investigated as potential inhibitors of the NET formation process since reactive oxygen species (ROS) directly influence the steps of NET production^16–18^.

Resveratrol (RSV) is a polyphenol stilbenoid characterized by two phenol rings joined by an ethylene bridge^19^. It is found in various plant species and human food sources such as fruits, vegetables, and chocolate^20^. RSV can exert its effects by acting as an agonist for different receptors^21,22^ or by freely crossing the plasma membrane^23,24^ to modulate the response of target molecules located in the cytoplasm, nucleus, and plasma membrane. This versatility allows RSV to trigger biological effects through various signaling pathways^25–29^. Notably, RSV is highly safe, as it does not exhibit significant side effects even when administered in single or divided doses of up to 1 g/day^30,31^. RSV is widely recognized for its potent antioxidant properties, specifically in scavenging superoxide, hydrogen peroxide, nitrite peroxide, and hydroxyl radicals^32^. Additionally, RSV has been observed to possess cardioprotective, neuroprotective, microbicidal, and anti-inflammatory effects^33–36^.

Given the critical role played by reactive oxygen species (ROS) in the release of NETs^11^, our study aimed to investigate the potential of RSV to modulate this release induced by classical and parasitic stimuli. In our research, we observed that RSV effectively reduces cellular hydrogen peroxide levels and alters the localization of NE, leading to a decrease in the release of NETs.

## Results

### Resveratrol inhibits the release of NETs induced by different stimuli

**Figure 1:**
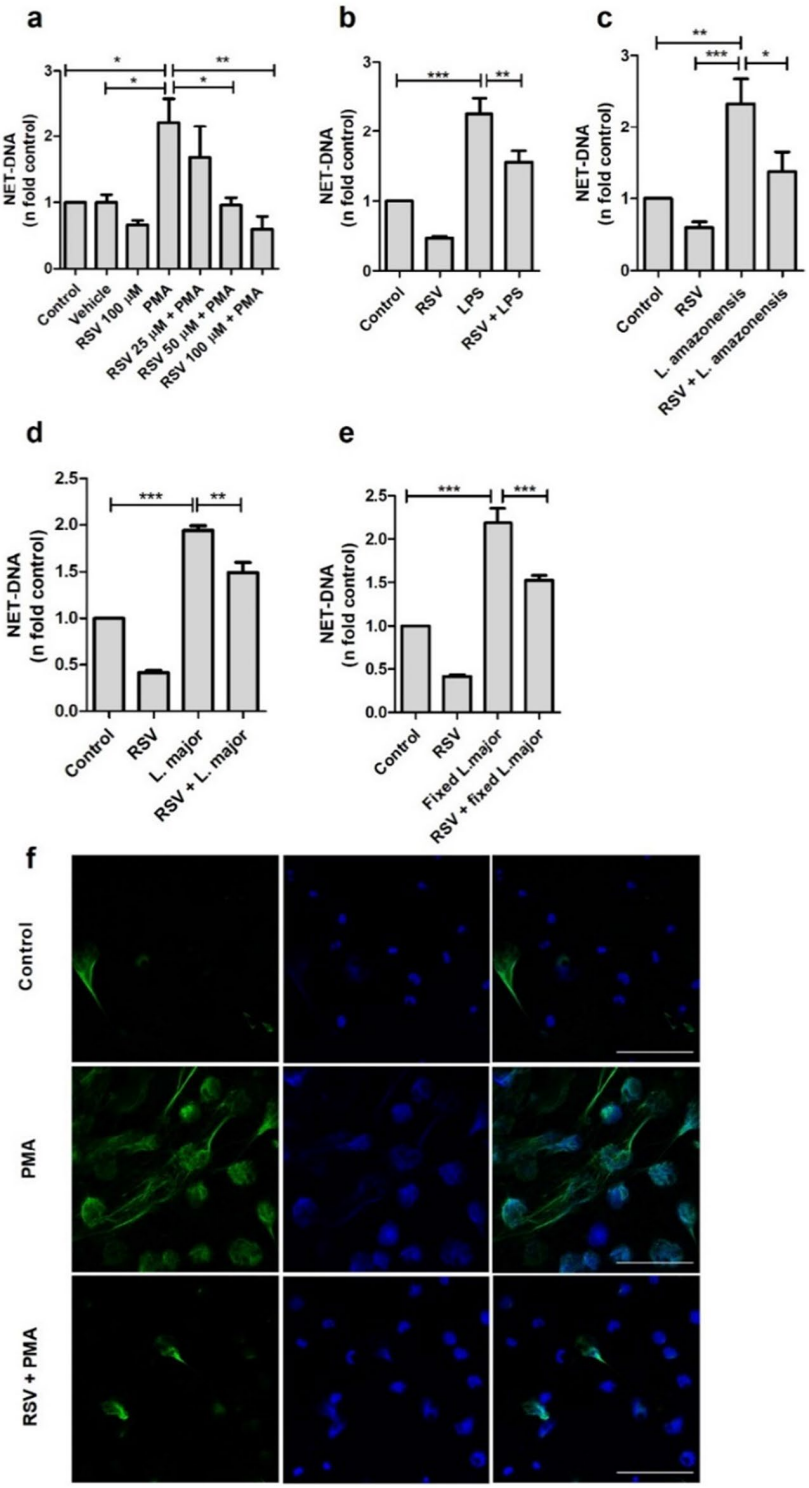
Resveratrol decreases the release of NETs by neutrophils stimulated with PMA, LPS, *Leishmania amazonensis*, and *L. major*. Neutrophils (5 × 10^4^/well) were pretreated with different concentrations of resveratrol (RSV) for 30 min and then stimulated with (**a**) 100 nM PMA, (**b**) 10 µg/ml LPS, (**c**) *Leishmania amazonensis*, (**d**) *L. major* or (**e**) fixed *L. major (*1NØ: 1 *Leishmania* ratio) for 4 h. The amount of NET-DNA in the supernatant was quantified using PicoGreen and normalized to the control. Results expressed as n fold related to control, mean ± SEM of at least 3 donors. (**f**) Immunofluorescence of neutrophils labeled with anti-elastase antibody (green) and DAPI (blue). Bar: 50 µm. *p < 0.05; **p < 0.001; ***p < 0.0001.

Human neutrophils were pretreated with different concentrations of RSV and subsequently stimulated with PMA to investigate its inhibitory potential. Our findings revealed that pretreatment with 50 and 100 µM of RSV effectively inhibited the release of PMA-induced NETs (Fig. 1a) while maintaining cell viability (Supplementary Figure S1a). We have selected 50 µM of resveratrol, the lowest concentration that exhibited an inhibitory effect, for subsequent experiments. Due to inter-donor variation, the results were presented as fold changes, whereas the raw data depicting the variation in NETs released from each donor were included in Supplementary Figure S1b.

Since PMA is a non-biological stimulus, we sought to determine whether RSV could inhibit NETs triggered by other well-characterized stimuli. Thus, we tested LPS and found that RSV also reduced NETs stimulated by this bacterial molecule (Fig. 1b) as well as by *Leishmania amazonensis* (Fig. 1c) and *Leishmania major* (Fig. 1d). In a previous study by our group^36^, it was evidenced the leishmanicidal activity of RSV. To ensure that the compound was not only acting on *Leishmania*, but on neutrophils, fixed promastigotes of *L. major* were used. Our observations revealed that even after the fixation process, the parasites still could prompt neutrophils to release NETs (Supplementary Figure S2), an effect significantly reduced following pre-treatment with RSV (Fig. 1e). Interestingly, RSV decreased even the basal release of NETs (Fig. 1a-e).

Aiming to strengthen our data, we analyzed the morphology and characterized the presence of elastase, a known enzyme associated with the DNA scaffold of NETs, in addition to measuring free DNA with picogreen. To visually illustrate the reduction in NETs caused by RSV treatment, neutrophils were labeled with an anti-neutrophil elastase antibody and stained with the DNA intercalating agent DAPI. The images validated our quantitative results, indicating that control neutrophils or those pre-treated with RSV and stimulated with PMA exhibited less chromatin decondensation and NETs released than neutrophils stimulated only with PMA (Fig. 1f).

Collectively, these data demonstrate that RSV can decrease the release of NETs induced by PMA, LPS, and *Leishmania* stimuli.

### The decrease of NETs by resveratrol is independent of ROS production

**Figure 2:**
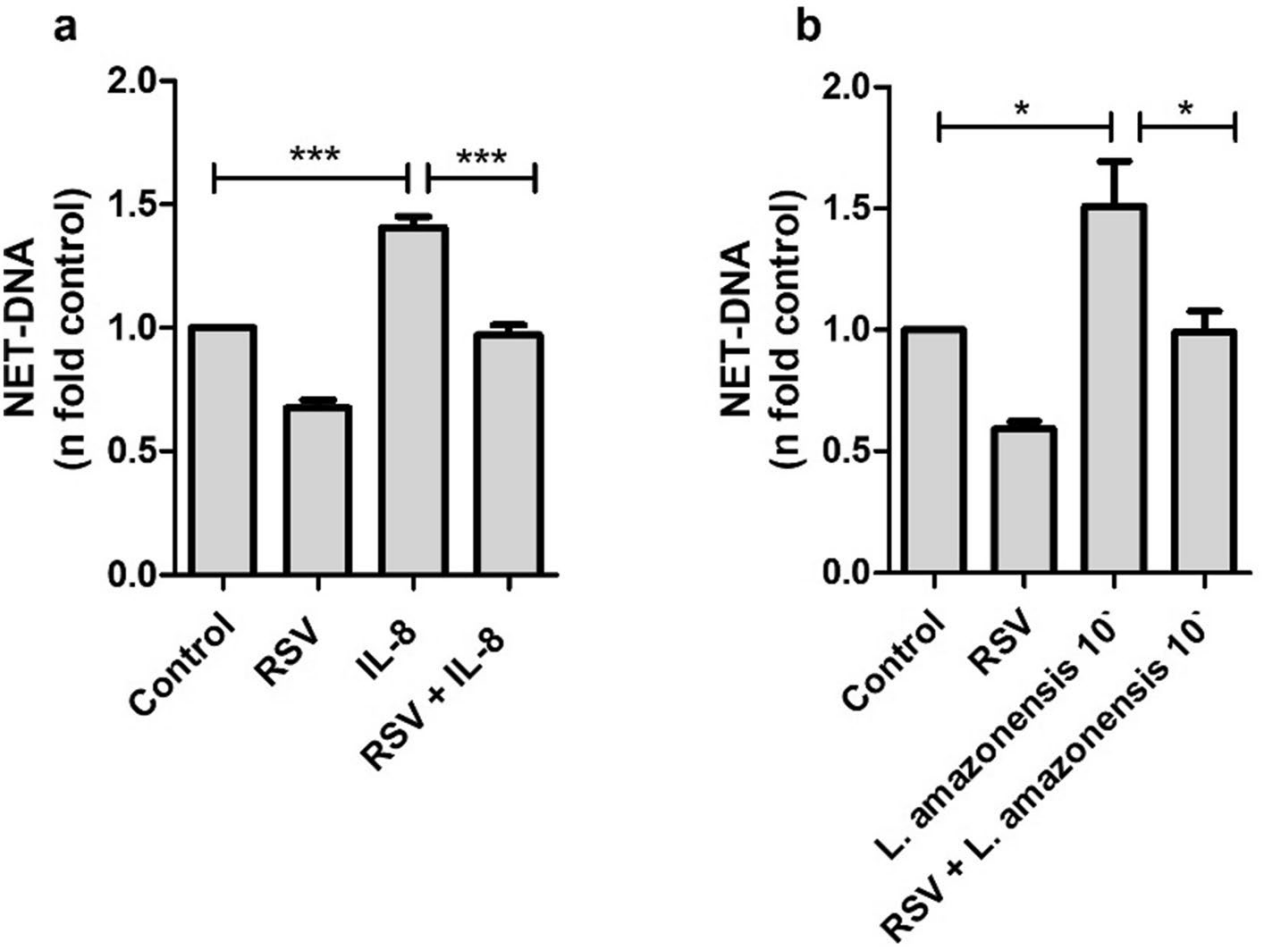
Resveratrol inhibits the release of NETs induced by ROS-independent stimuli. Neutrophils (5 × 10^4^/well) pretreated with 50 µM of resveratrol (RSV) for 30 min were then stimulated for 4 h with (**a**) 100 ng/ml IL-8 or (**b**) 10 min with *Leishmania amazonensis* promastigotes (1NØ: 1 *Leishmania* ratio). The amount of NET-DNA in the supernatants was quantified using PicoGreen. Results shown as n-fold related to control, expressed as mean ± SEM of 4 (**a**) and 6 (**b**) donors. *p < 0.05; ***p < 0.0001.

Previous studies have shown that reactive oxygen species (ROS) may or may not be triggered to release NETs depending on the stimulus^37,38^. Considering the antioxidant properties of RSV, it is worth seeing if its ability to inhibit the release of NETs also applies to stimuli that do not generate ROS. To address this question, we pretreated neutrophils with RSV and stimulated them with IL-8 and *Leishmania amazonensis* promastigotes, which triggered NETs release by the early/rapid mechanism independently of ROS in 10 min stimulation^39^. Remarkably, we observed that RSV efficiently inhibited the release of NETs in response to IL-8 (Fig. 2a) and promastigotes stimulation (Fig. 2b).

### A2A and A2B adenosine receptors do not participate in the decrease of NETs by resveratrol

**Figure 3:**
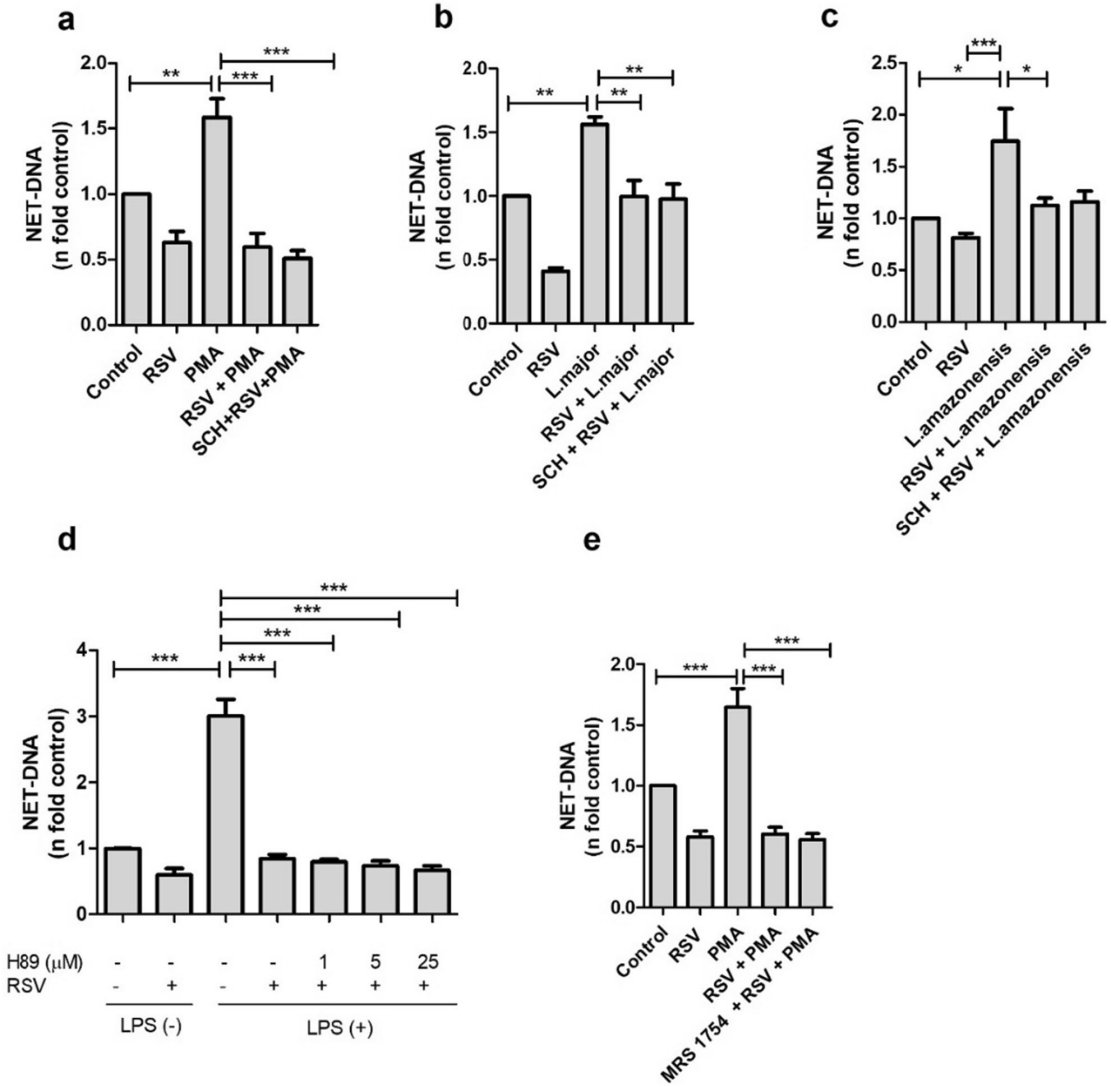
The effect of resveratrol on the modulation of NETs is not related to signaling through A2A, A2B adenosine receptors, or PKA. Neutrophils (5 × 10^4^/well) were pretreated with 100 nM of the selective antagonists to A2A receptor (SCH 58261) or A2B receptor (MRS 1754) for 15 min, followed by treatment with 50 µM of RSV for 30 min and stimulation with (**a**, **e**) 100 nM PMA, (**b**) *Leishmania major* for 4 h or (**c**) *L. amazonensis* for 10 min (1NØ: 1 *Leishmania* ratio). (**d**) Neutrophils were pretreated with RSV and PKA inhibitor for 30 min and then stimulated with 10 µg/ml of LPS. NET-DNA quantification was performed in the supernatant by PicoGreen. Results are shown as n-fold related to control, expressed as mean ± SEM of 3 (**a**, **b**), 7 (**c**), 4 (**d**), and 5 (**e**) donors. *p < 0.05; **p < 0.001; ***p < 0.0001.

Next, we investigated cellular targets through which RSV modulates the release of NETs. Previous studies have indicated that RSV can function as an agonist of the A2A adenosine receptor^22^, and this receptor has been implicated in the release of NETs induced by PMA^40^. To explore the participation of the A2A adenosine receptor, we pretreated neutrophils with its selective antagonist SCH58261. Our results showed that treatment with the antagonist did not reverse the NET-lowering effect of RSV in neutrophils stimulated by PMA (Fig. 3a), *Leishmania major* (Fig. 3b), or *L. amazonensis* (Fig. 3c).

Further, we conducted experiments using different concentrations of H89, a PKA inhibitor, one of the downstream targets of the A2A adenosine receptor. Again, our findings demonstrated that PKA inhibition did not reverse the effect of RSV on the modulation of NETs induced by LPS (Fig. 3d).

Previous research has suggested the involvement of the A2B adenosine receptor in inhibiting PMA-induced NETs by adenosine^40^. To investigate whether RSV acts on the same receptor, we used a selective A2B receptor antagonist, MRS1754, but blocking the receptor did not reverse the effect of RSV (Fig. 3e).

Our data indicates that RSV does not act through adenosine A2A receptors to reduce the release of NETs stimulated by PMA, LPS, and *Leishmania*. Additionally, we did not observe the involvement of the A2B receptor in PMA-induced NET extrusion or the participation of PKA in LPS-stimulated NETs.

### Modulation of the hydrogen peroxide-MPO axis by resveratrol

**Figure 4:**
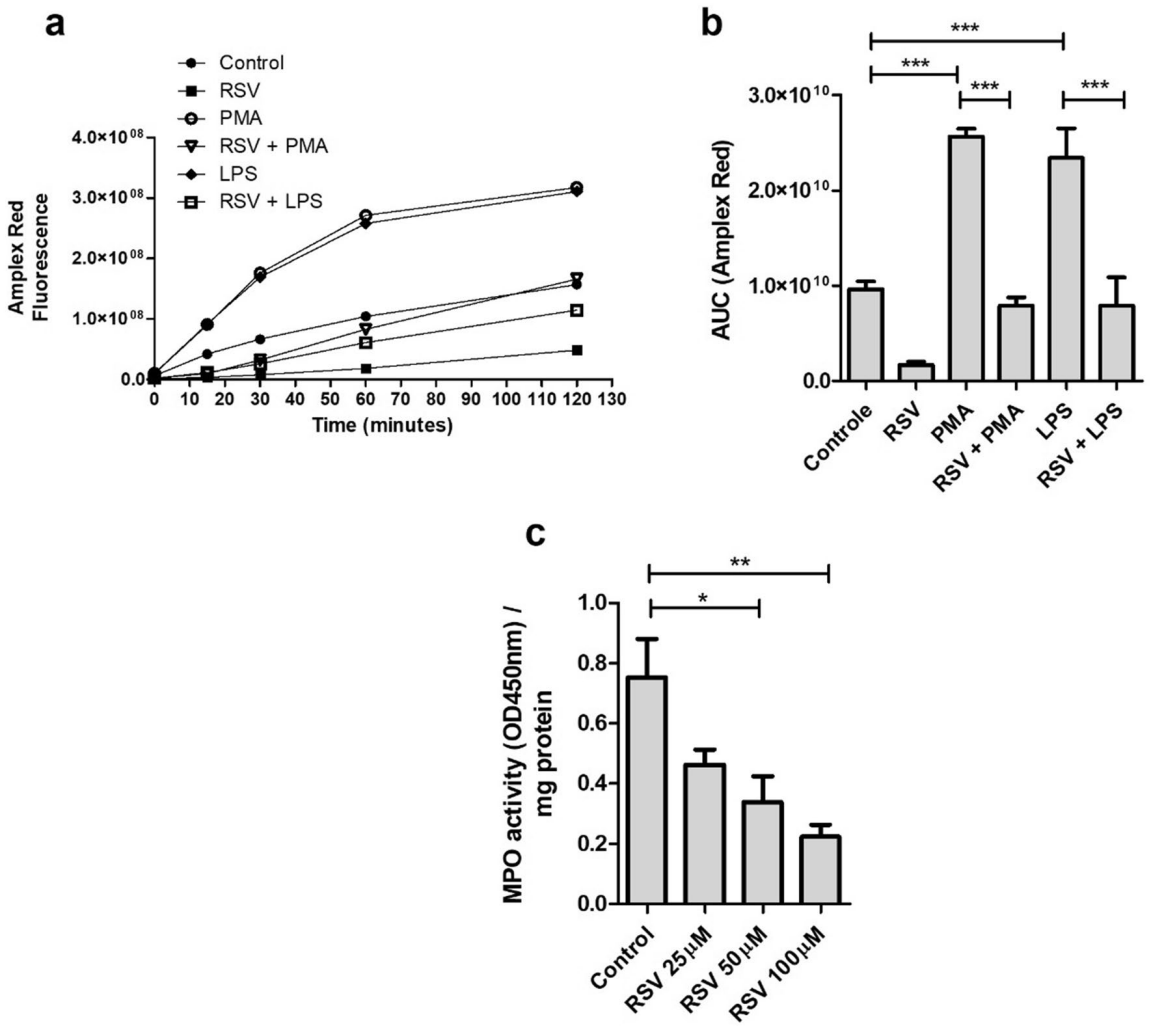
Modulation of the H_2_O_2_-MPO axis in human neutrophils treated with resveratrol. (**a**) Kinetics of hydrogen peroxide detection after pretreating neutrophils (5 × 10^5^/well) with 50 µM of RSV for 30 min followed by the addition of Amplex Red probe and stimulated or not with 100 nM of PMA or 10 µg/ml of LPS. Result from a representative donor out of 4 tested. (**b**) Area under the curve (AUC) of the amount of ROS released by neutrophils stimulated from 4 donors. (**c**) The activity of intracellular MPO was quantified in neutrophils upon the 1 h addition of RSV. Results shown as Amplex Red fluorescence and MPO activity/mg protein, expressed as mean ± SEM of 4 (**b**, **c**) donors. *p < 0.05; **p < 0.001, ***p < 0.0001.

Another potential target for the RSV action is hydrogen peroxide (H_2_O_2_). It has been previously demonstrated that H_2_O_2_, among the components comprising cellular ROS, plays a crucial role in inducing NETs by facilitating the release of elastase from the azurophilic granule and enabling its translocation to the nucleus, thus promoting chromatin decondensation^41^. To investigate the potential relationship between RSV and H_2_O_2_ in the modulation of NETs release, we initially measured total ROS production in PMA-stimulated neutrophils to confirm the antioxidant capacity of RSV. According to our findings, RSV significantly inhibits the ROS production induced by PMA and reduces it to levels comparable to that of DPI (Supplementary Figure S3). Then, we examined whether RSV directly affected the cellular levels of H_2_O_2_. Our data revealed a significant reduction in H_2_O_2_ levels in RSV-pretreated neutrophils stimulated with PMA or LPS in the kinetic assay (Fig. 4a,b). Considering that hydrogen peroxide is one of the substrates for MPO, we treated human neutrophils with different concentrations of RSV and subsequently assessed the MPO enzymatic activity. We observed a dose-dependent decrease in MPO activity with 50 and 100 μM of RSV (Fig. 4c), suggesting that H_2_O_2_ may contribute to the NETs release inhibition in neutrophils stimulated by PMA and LPS.

### The localization of neutrophil elastase is modulated by resveratrol

**Figure 5:**
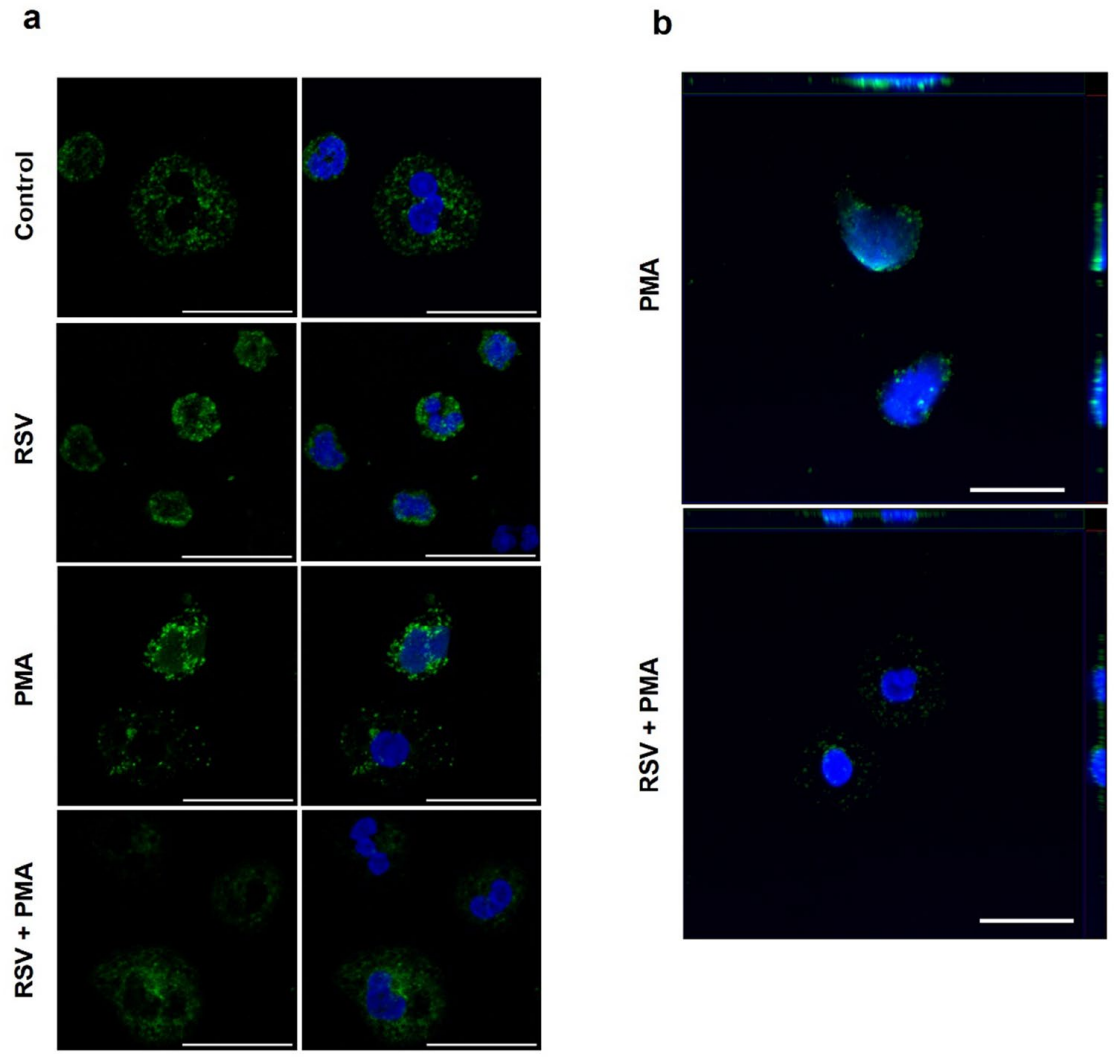
Resveratrol modulates the localization of neutrophil elastase. Adherent neutrophils were pretreated or not with 50 μM RSV for 30 min and subsequently stimulated with 100 nM PMA for (**a**) 1 h or (**b**) 2 h. Cells were stained with anti-elastase antibody (green) and DAPI (blue). (**a**) Maximum intensity projection (MIP) of 12 optical slices along the Z axis. (**b**) Orthogonal view of 9 optical slices along the Z axis. Bar: 20 μm.

It is well established that oxidation mediated by hydrogen peroxide plays a crucial role in disassembling proteins from the microbicidal protein complex within azurophilic granules, leading to the release of NETs^41^. A change in the cellular redox state facilitates the dissociation of MPO from NE within azurophilic granules, activating its proteolytic function. Activated NE translocate from the granule to the nucleus, cleaving the tails of histones H1, H2B, and H4, initiating chromatin decondensation^11,41^. Based on our data, which demonstrated the role of RSV in reducing hydrogen peroxide levels induced by PMA and inhibiting NETs release, we hypothesized that RSV may alter the localization of NE. Thus, we treated neutrophils with PMA or RSV + PMA and followed elastase location by immunofluorescence. Our results revealed that 1 h after stimulating neutrophils with PMA, nuclear staining of neutrophil elastase was already visible (Fig. 5a). This staining intensity increased after two hours of stimulation (Fig. 5b). In contrast, neutrophils pre-treated with RSV and stimulated with PMA showed little to no nuclear elastase staining within the first 2 h of stimulation (Fig. 5b), supporting our hypothesis that the decrease in hydrogen peroxide levels modulates the localization of elastase and, consequently, the release of NETs. Additionally, we stimulated neutrophils with *Leishmania amazonensis* and LPS. Both stimuli corroborated the data obtained with PMA stimulation, demonstrating the effectiveness of the antioxidant in modulating neutrophil elastase localization (Supplementary Figure S4).

## Discussion

In 2004, the first article was published describing NETs as an innate immune mechanism responsible for trapping and killing pathogens^1^. Since then, it has been reported that multiple cell types can release NET-like structures. However, they can lead to pathophysiological mechanisms if produced excessively or not efficiently removed^42^. As a result, new therapeutic strategies have been investigated to inhibit or delay the release of NETs^43^.

This study examined RSV's impact on various NET release stimuli. RSV is a polyphenol commonly found in various fruits and vegetables, renowned for its potent antioxidant action^20^ and anti-inflammatory, cardio, and neuroprotective effects^44^. We report here that RSV reduces the production of NETs induced by PMA, LPS, and *Leishmania* without affecting neutrophil viability.

RSV has been proven safe in several clinical trials, with single or divided doses of up to 1 g/day. Healthy subjects tolerated doses of up to 5 g/day without any adverse effects, whereas liver disease patients exhibited symptoms such as nausea, vomiting, and diarrhea after administration of 2.5 g/day of the compound^30,31^. After taking a single oral dose of RSV, the bioavailability in the blood increases for up to 1 h, and the plasma pharmacokinetic parameters peak at 0.6 μM for a 5 mg dose and 137 μM for a 1 g dose. After 24 h, the values ranged from 0.05–0.12 μM to 12–18 μM, respectively^45^.

RSV was also highly effective in reducing NETs generated by inducers that do not produce ROS in neutrophils, such as the early/rapid NET extrusion by *Leishmania amazonensis*^39^. Contradictory results reported that IL-8 is unable to induce ROS in neutrophils^46,47^, although others have shown the production of this mediator by IL-8 stimulated neutrophils^48^. We used IL-8 as a ROS-independent NET stimulus since, in our conditions, we did not detect ROS in IL-8-stimulated neutrophils^49^. The capacity of RSV to inhibit NET formation indicates its broad capacity to act on different NET stimuli. In addition to inhibiting NET generation, it is hypothesized that RSV, due to the reduction of copper with the concomitant formation of reactive oxygen species, could cleave the DNA scaffold of NETs^50^.

While NETs play a crucial role in host defense, excessive production can have detrimental effects on the body due to the disruptive nature of their components, which can interfere with cell junctions, compromise the integrity of eukaryotic cells, and impair membrane function^38^. During the COVID-19 pandemic, patients infected with the SARS-CoV-2 exhibited elevated levels of NETs in their plasma, bronchoalveolar aspirate, and lungs^3,51^. Infection with this virus triggers the secretion of IL-8, leading to the recruitment of neutrophils near pulmonary capillaries, where they release toxic NETs that harm pulmonary cells^3^. This process also involves platelet aggregation and the secretion of adhesion molecules by the endothelium, contributing to vessel occlusion and respiratory failure, thereby exacerbating the severity of the disease^51^. Consequently, it is of utmost importance to identify safe drugs that can inhibit or reduce the release of NETs. Recent studies have shown that RSV significantly decreases the extrusion of spontaneous NET release in neutrophils obtained from COVID-19 patients and after stimulation of these neutrophils with PMA^52^.

To better understand the mechanism by which RSV inhibits the release of NETs, we investigated the role of A2A, the most abundant adenosine receptor in the neutrophil membrane^40^. Previous studies have demonstrated that RSV acts as a non-selective agonist of the A2A receptor, and blocking the receptor with an A2A antagonist reverses the adenosine-mediated inhibition of NETs. Additionally, RSV non-selectively inhibits phosphodiesterase 1, 3, and 4, increasing cellular cAMP^26^. Interestingly, it has been shown that *Bordetella pertussis* exploits the mechanism of increased cAMP to inhibit NET production and evade NET-ensnaring^53^. However, our results showed that treatment with A2A and A2B receptor antagonists or a PKA inhibitor, a downstream target of cAMP, failed to reverse the inhibitory effect of RSV on NETs induced by any of the stimuli tested.

One of the fundamental requirements for NE to leave the granule and initiate the process of chromatin decondensation is the presence of H_2_O_2_, which is responsible for breaking down the azurosome protein complex, allowing the release of NE^41^. Our findings demonstrate that RSV inhibits the PMA- and LPS-induced increase of H_2_O_2_. Moreover, RSV-treated neutrophils show levels of H_2_O_2_ below baseline, which explains the lower NET release we observed in the RSV condition compared to the control. Additionally, H_2_O_2_ is a substrate for MPO, which reacts with chlorine to produce hypochlorous acid and ROS^28^. We observed that RSV decreases MPO activity. Furthermore, RSV has been described as acting directly on the MPO enzyme in horse neutrophils, preventing substrate access to the enzymatic active site^54^. With the reduction of the H_2_O_2_-MPO axis induced by RSV and the resulting decrease in NETs, we hypothesize that this effect may be attributed to the inhibition of NE migration from the granule to the nucleus. It is already known that NE reaches the nucleus of neutrophils rapidly following PMA stimulation, typically observed after 30 min^11^. In our study, we investigated the nuclear localization of NE after 1 and 2 h of PMA, LPS, and *Leishmania* stimulation and confirmed the nuclear migration of NE. However, in the RSV condition, we observed limited NE nuclear migration at either time analyzed. The observed staining was cytoplasmic and exhibited a dotted pattern, suggesting that NE may be localized in neutrophilic granules. These findings indicate that RSV effectively regulated NE distribution on the PMA-stimulated human neutrophils, decreasing NET release.

Our findings showed that RSV may contribute to decreasing excessive NETs production, pointing RSV as a candidate compound with the potential for mitigating NET-derived pathologies.

## Conclusion

Our research shows that RSV effectively reduces the release of NETs caused by PMA, LPS, IL-8, and *Leishmania *in vitro, independent of the A2A or A2B adenosine receptors and the PKA pathway. RSV also significantly decreased H_2_O_2_ levels and changed NE localization in stimulated neutrophils. Further investigations are warranted to validate RSV as a potential candidate for testing in NET-exacerbated diseases.

## Methods

### Ethics statement

Experimental procedures involving cells from healthy donors were performed with blood samples obtained after written informed consent and approved by the Research Ethics Committee (Hospital Universitário Clementino Fraga Filho, UFRJ, Brazil, protocol number: 4261015400005257). All assays were performed in accordance with the committee's guidelines and regulations.

### Neutrophil purification

Peripheral blood collected from healthy donors was used to isolate neutrophils by density gradient centrifugation, as described^10^. The neutrophils were washed, resuspended in RPMI, and used throughout.

### Parasites

*Leishmania amazonensis* (WHOM/BR/Josefa) and *Leishmania major* (MHOM/IL/80/Friedlin) were grown in Schneider medium (Sigma-Aldrich) supplemented with 10% fetal calf serum and 1% penicillin–streptomycin at 26 °C The stationary phase promastigotes were washed three times with PBS (1045 g, 10 min, 4 °C) and, resuspended in medium RPMI 1640. *L. major* promastigotes were washed with PBS and incubated for 15 min with PNA lectin (Sigma) to separate the metacyclic form. 4% formaldehyde was used for parasite fixation, followed by two washes with PBS.

### Resveratrol assay

Purified neutrophils (5 × 10^4^ cells/well) were cultured in 96 wells and kept at 37 °C, 5% CO_2_ for 20 min for adhesion. Next, the cells were pretreated with different concentrations (25, 50, and 100 µM) of resveratrol (Sigma-Aldrich) for 30 min. Subsequently, the neutrophils were stimulated with PMA (100 nM; Sigma-Aldrich), delipidated LPS (10 μg/mL; Sigma-Aldrich), IL-8 (100 ng/mL; (PeproTech), and with *Leishmania major* (parasite: cell, 1:1) or *Leishmania amazonensis* (1:1) and incubated as above. All experiments were performed in RPMI 1640 medium without serum, and DMSO was used as a vehicle. Adhered neutrophils were pretreated with 100 nM of selective A2A receptor antagonist (SCH58261, Sigma-Aldrich) or with 100 nM selective A2B receptor antagonist (MRS 1754, Sigma-Aldrich) for 15 min at 37 °C, 5% CO_2_ prior the resveratrol treatment. For the PKA experiment, the H89 inhibitor (1, 5 and 25 µM; Sigma-Aldrich) was added to neutrophils along with RSV treatment for 30 min. Subsequently, the cells were stimulated with LPS.

### NET quantification

NETs were measured in the culture supernatants after centrifugation at 400 g for 5 min at room temperature. For experiments involving *Leishmania*, supernatants were collected and centrifuged at 2700 g for 10 min to sediment the parasites. According to the manufacturer's instructions, the DNA assay was performed in opaque 96-well plates using the PicoGreen dsDNA kit (Invitrogen Life Technologies). Sample readings were performed on the SpectraMax Paradigm device (Molecular Devices) at 485/538 nm (excitation/emission).

### Myeloperoxidase (MPO) activity assay

Neutrophils (5.6 × 10^5^/well) adhered in 24 wells were treated with 25, 50, or 100 μM of resveratrol. After 1 h, the supernatant was discarded, and cells lysed with NETN buffer (100 μL/well of 250 mM NaCl, 5 mM EDTA—pH 8.0, 50 mM Tris-HCl—pH 8.0, Igepal-CA630 0.5% (Sigma), 1% protease inhibitor (Sigma), and NaF (50 μM; Sigma) at 4 °C for 10 min. MPO activity was assayed (25 μL) of the lysate with (25 μL) of TMB-H_2_O_2_ substrate (ThermoFisher) under stirring for 10 min, and the reaction stopped with 1 M H_2_SO_4_ and read at 450 nm. Protein concentration was determined using the Bradford method.

### Quantification of hydrogen peroxide production

Neutrophils (5 × 10^5^/well) were pretreated or not with 50 μM resveratrol for 30 min. Then, 100 nM PMA was added with the Amplex Red fluorescent probe (5 μM, ThermoFisher) and Horseradish Peroxidase (2 units/mL, Sigma). Kinetics of up to 120 min was performed at 530/590 nm (excitation/emission).

### Immunofluorescence

Neutrophils (2 × 10^5^ cells/well) plated on coverslips (Knittel) coated with 0.001% poly-L-lysine (Sigma) were pretreated or not, with 50 μM resveratrol for 30 min and stimulated with 100 nM PMA, 10 µg/ml LPS or *Leishmania amazonensis* (1:1, neutrophil: parasite ratio) for 1, 2 or 4 h. Cells were fixed with 4% formaldehyde. Coverslips were washed three times with PBS, blocked with 1% BSA (Sigma), and incubated with anti-neutrophil elastase antibody (1:500; Calbiochem) diluted in the blocking solution, followed by anti-rabbit Alexa Fluor 488 (1:800; ThermoFisher), and DNA stain (DAPI, Sigma). Coverslips were washed and mounted with ProLong Gold Antifade with DAPI (ThermoFisher). For intracellular labeling, 0.1% of Triton X-100 (Sigma) was used for cell permeabilization. The confocal microscopes Elyra PS.1 with SR-SIM (Zeiss) and Leica TCS SPE (LAS X 3.1.2.16221) and Ti2-U (Nikon) were used to take images.

### ROS production

Neutrophils (10^5^ cells/well) were preincubated with DPI (10 μM, diphenylene iodonium chloride, Sigma) or 50 μM resveratrol for 30 min at 37 °C, 5% CO_2_. Afterward, they were treated with 100 nM PMA and a 1.2 µM dihydrorhodamine 123 fluorescent probe (DHR, Sigma). The fluorescence intensity was measured for 2 h at 500/570 nm (excitation/emission). These experiments were performed in RPMI 1640 medium without phenol.

### Viability assay

Neutrophil culture supernatants were centrifuged at 400 g for 5 min, and cell viability was performed using the CytoTox kit 96 Non-Radioactive Cytotoxicity Assay (Promega), according to the manufacturer's instructions and read at 490 nm.

### Statistical analysis

The data were analyzed by ANOVA and Tukey post-test, using GraphPad Prism version 5.00. The experiments were performed in duplicates at least three independent times. *p* < 0.05 was considered significant.

### Supplementary Information

Supplementary Information.

## Data Availability

The data supporting this study's findings are available from the corresponding author upon reasonable request.
