# Supplementary material for: Resveratrol prevents the release of neutrophil extracellular traps (NETs) by controlling hydrogen peroxide levels and nuclear elastase migration
Source: Sci Rep. 2024 Apr 20;14:9107. doi: 10.1038/s41598-024-59854-2 (PMC11032324; doi:10.1038/s41598-024-59854-2)
Supplement: Supplementary file 1 — Supplementary Information. [file 41598_2024_59854_MOESM1_ESM.docx]

**Supplementary Information**

**Resveratrol prevents the release of neutrophils extracellular trap (NETs) by controlling hydrogen peroxide levels and elastase migration.**

Thayana Roberta Ferreira de Mattos^1^, Marcos Antônio Formiga Júnior^1^, Elvira Maria Saraiva^1^*


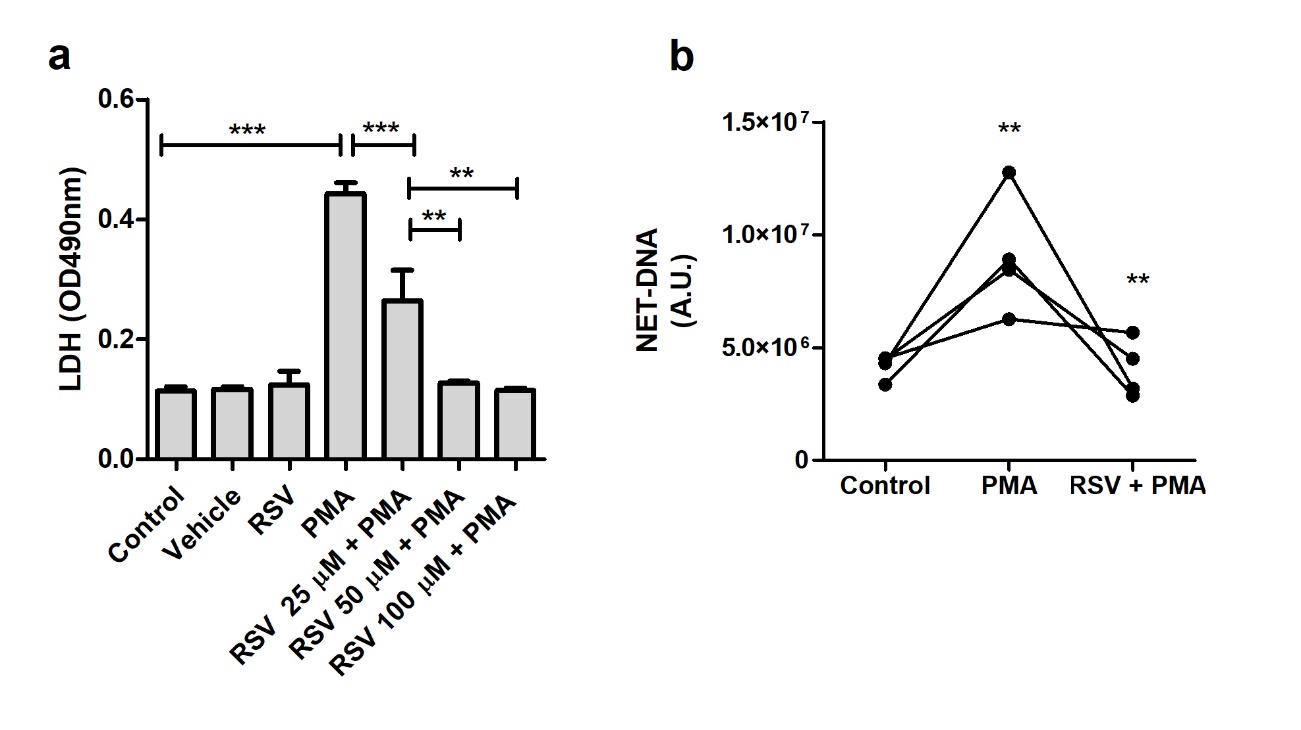


**FIGURE S1. Resveratrol prevents the death and the release of NETs by PMA-stimulated human neutrophils.** Neutrophils (5x10^4^/well) were pretreated with the indicated RSV concentrations for 30 min and then stimulated with 100 nM of PMA. (**a**) Results express as Mean ± SEM of neutrophils viability measured by LDH release of 4 donors. (**b**) Donor-to-donor variation in NET release. AU: arbitrary units. **p<0.001; ***p<0.0001.


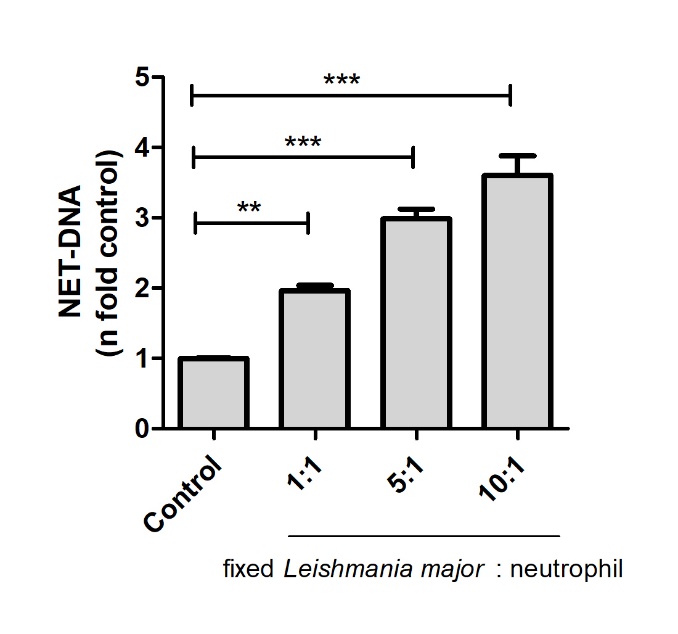


**FIGURE S2. Fixed *Leishmania major* induces the release of NETs in human neutrophils.** Neutrophils (5x10^4^/well) were stimulated with different proportions of fixed *Leishmania major* (4% formaldehyde) for 4 h. The amount of NET-DNA was measured in the supernatant by Picogreen. Results shown as n fold related to control, expressed as mean ± SEM of 4 donors. *p<0.05; **p<0.001; ***p<0.0001.


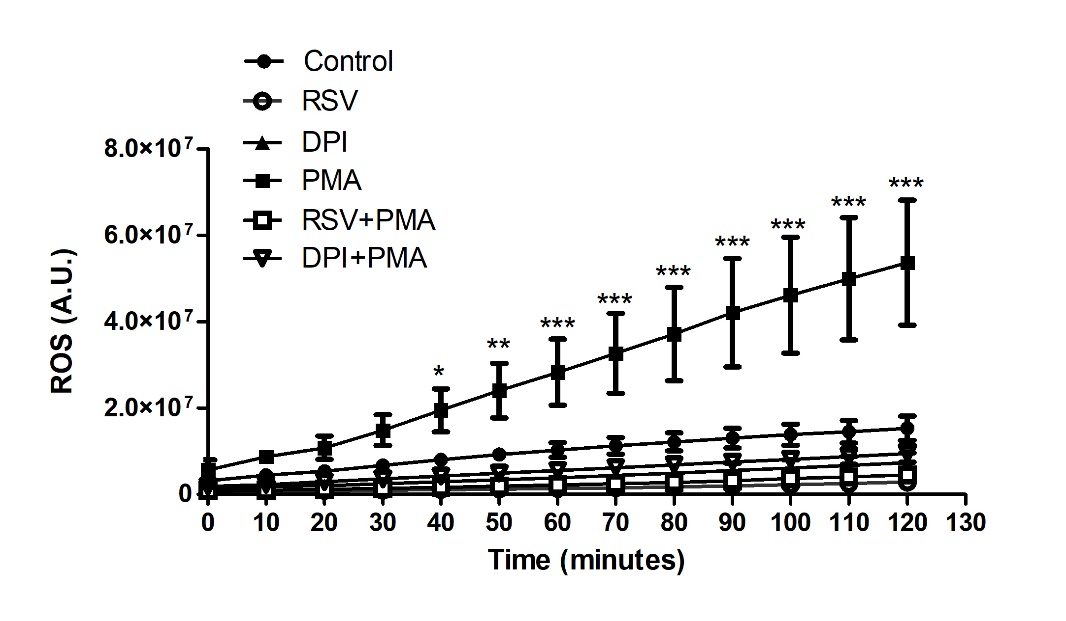


**FIGURE S3. Resveratrol (RSV) inhibits reactive oxygen species (ROS) in PMA-stimulated neutrophils.** The amount of ROS measured by the DHR 123 probe after neutrophils were pretreated with 50 µM of resveratrol or 10 µM of DPI and subsequently stimulated with 100 nM of PMA. Results are shown as ROS arbitrary units (A.U.) mean ± SEM of 13 donors. *p<0.05; **p<0.001; ***p<0.0001.

**
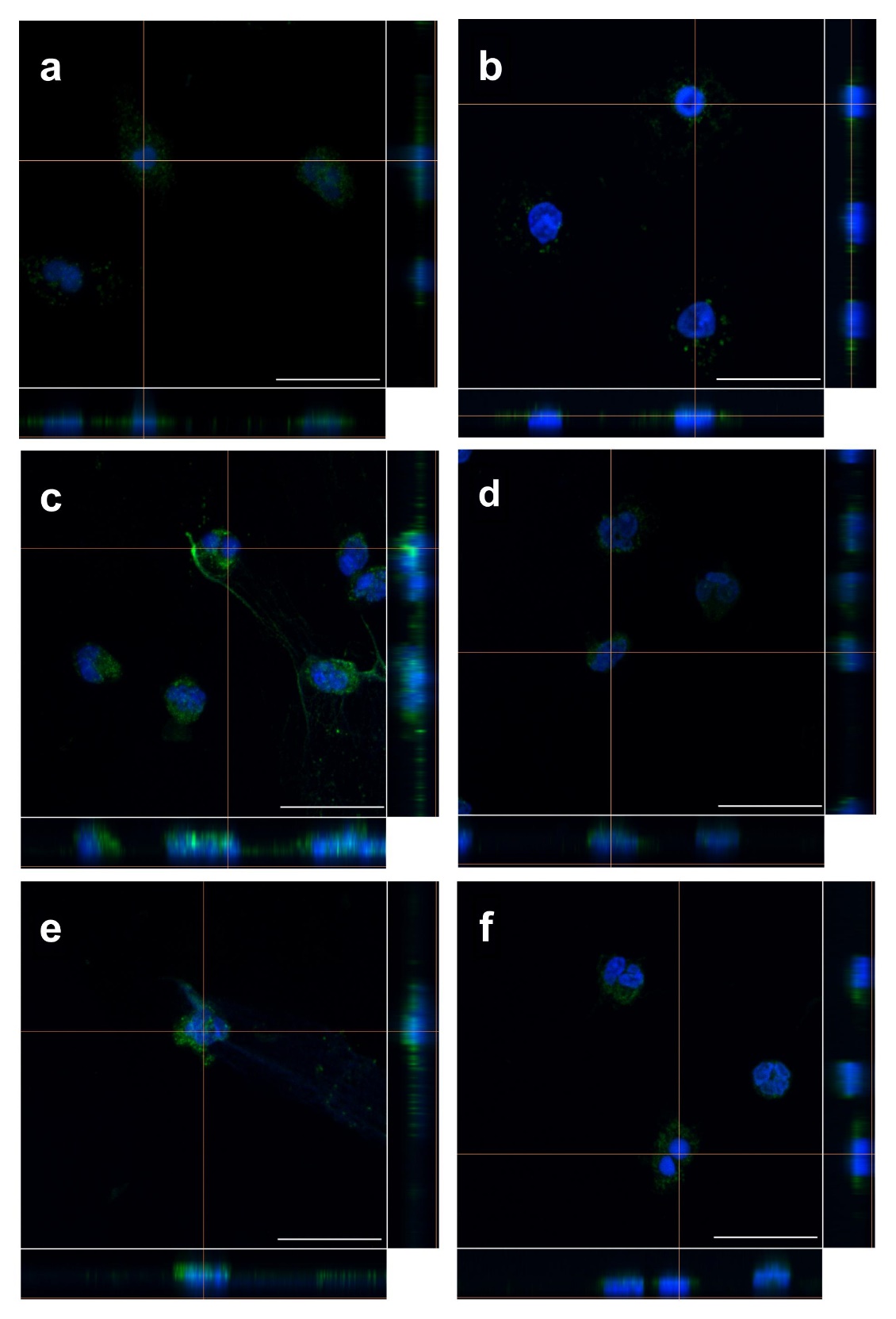
FIGURE S4. Resveratrol modulates the localization of neutrophil elastase in LPS- and *Leishmania*-stimulated neutrophils.** Adherent neutrophils (2x10^5^ cells) were pretreated or not with 50 μM RSV for 30 min and subsequently stimulated for 2 h with (**c**) 10 µg/ml LPS or (**e**) *Leishmania amazonensis* (MOI 1). Cells were stained with anti-elastase antibody (green) and DAPI (blue). Orthogonal view of 7 optical slices along the Z axis. **(a)** unstimulated control; **(b)** RSV; **(d)** RSV + LPS; **(e)** *L. amazonensis* (MOI 1), and **(f)** RSV + *Leishmania*. Bars: 20μm.
